# Synergistic effects of BET and MEK inhibitors promote regression of anaplastic thyroid tumors

**DOI:** 10.18632/oncotarget.26253

**Published:** 2018-10-23

**Authors:** Xuguang Zhu, Erik Holmsen, Sunmi Park, Mark C. Willingham, Jun Qi, Sheue-yann Cheng

**Affiliations:** ^1^ Laboratory of Molecular Biology, Center for Cancer Research, National Cancer Institute, National Institutes of Health, Bethesda, MD, USA; ^2^ Dana Farber Cancer Institute, Harvard Medical School, Boston, MA, USA

**Keywords:** tumorigenesis, anaplastic thyroid cancer, bromodomain and extraterminal protein inhibitor, JQ1, trametinib

## Abstract

Anaplastic thyroid cancer (ATC) is an aggressive malignancy with limited options for treatment. Targeting epigenetic modifications via interfering with the interaction between the bromodomain and extra-terminal domain (BET) proteins and acetylated histones by using BET inhibitors (e.g., JQ1) has shown some efficacy in thyroid cancer. To improve the efficacy, an inhibitor of MEK, trametinib, was tested together with JQ1 as a combined treatment via cell-based approaches and xenograft studies. We examined the effects of combined treatment of JQ1 and trametinib on the proliferation of human ATC cell lines (THJ-11T and THJ-16) *in vitro*. We further evaluated the effects of the combined treatment on tumor development *in vivo* using mouse xenograft models. We elucidated the underlying molecular pathways affected by double treatment. We showed that the combined treatment totally blocked proliferation, while either JQ1 or trametinib alone only had partial effects. Combined treatment suppressed MYC expression more than single treatment, resulting in decreased expression of pro-survival regulators and increased pro-apoptotic regulators to collaboratively induce apoptosis. In xenograft studies, single treatment only partially inhibited tumor growth, but the combined treatment inhbited tumor growth by >90%. The reduction of tumor growth was mediated by synergistic suppression of MYC, to affect apoptotic regulators to markedly promote tumor apoptosis. Combined treatment of BET and MEK-ERK inhibitors was more effective to treat ATC than single targeted treatment. Synergistic suppression of MYC transcription via collaborative actions on chromatin modifications suggested that targeting epigenetic modifications could provide novel treatment opportunities for ATC.

## INTRODUCTION

Anaplastic thyroid cancer (ATC) is one of the most aggressive malignancies. This cancer has poor prognosis and effective treatment modalities are very limited. Among the genetic and epigenetic abnormalities recently identified in ATC, MYC was uncovered as a critical oncogene in promoting the development and progression of thyroid cancer [1, 2]. MYC protein abundance was highly elevated in ATC tumors [1] and the increased MYC expression was associated with unfavorable prognosis [3, 4]. In a mouse model of ATC, increased expression of *Myc* was shown to promote thyroid cancer progression [5]. When *MYC* expression was blocked by antisense oligonucleotides, the proliferation of human thyroid cancer cell lines was inhibited [6]. These observations suggested that MYC could be a potential target for therapeutic intervention in ATC.

However, despite intensive research, targeting MYC protein itself has appeared to be an insurmountable challenge. No effective pharmacologic agents against MYC have yet to be identified. Recently, small-molecule compounds targeting epigenetic modifications have been utilized to modify *MYC* expression. Chromatin remodeling through histone acetylation is critical in the regulation of the gene expression in both normal and cancer cells [7]. Bromodomain and extraterminal domain (BET) family of proteins (e.g., BRD4) interact with acetylated lysine residues of histones to assemble chromatin complexes and transcription activators at specific promoter sites [8]. Selective small-molecule inhibitors, such as JQ1, block the interaction of BET proteins with acetylated histones on chromatin to alter transcription of affected genes [9]. JQ1 is a potent inhibitor of the *MYC* transcriptional program via attenuation of superenhancers [9]. Many reports have demonstrated the therapeutic efficacy of JQ1 in the treatment of acute lymphoblastic leukemia, acute myeloid leukemia, chronic lymphocytic leukemia, chronic myeloid leukemia, and some solid tumors [10–13].

Recently, the efficacy of JQ1 on thyroid cancer has also been evaluated in cell-based studies *in vitro* and in mouse models *in vivo*. JQ1 was shown to decrease MYC expression, arrest cell cycle progression, and suppress tumor growth *in vivo* [14, 15]. We also assessed the efficacy of JQ1 in a preclinical mouse model of ATC [16]. We found that JQ1 effectively suppressed MYC expression at the mRNA and protein levels. Further, JQ1 treatment significantly prolonged survival, inhibited tumor growth, and attenuated transcriptional programs critical for tumor cell proliferation [16]. However, intriguingly, we found that the effect of JQ1 was limited to the inhibition of tumor growth, and had no effect on tumor cell invasion and metastasis. These observations raised the possibility that thyroid tumor proliferation and invasion could be directed by separate cellular pathways. This hypothesis dictated the need to have a second agent effective in blocking the pathways involved in tumor invasion. We chose a MEK inhibitor, trametinib, on the basis that the MAPK-MEK signaling pathway is often over-activated in human ATC. Further, trametinib has been approved as a single-agent by the FDA for the treatment of patients with metastatic melanoma [17]. Further, trametinib enhances the suppression of JQ1-induced MYC expression and tumor growth in colorectal cancer [18, 19]. In the present study, we investigated the effects of the combined treatment of JQ1 and trametinib on the proliferation of human ATC cells *in vitro* and xenograft tumor growth *in vivo*. We found that the combination of the two inhibitors synergistically inhibited the recruitment of BET proteins to the promoter of the *MYC* gene to suppress the *MYC* transcription. MYC protein abundance in cells treated with double treatment was synergistically lower than with either single agent, leading to an enhanced inhibitory effect on tumor cell proliferation, tumor growth, and tumor cell invasion. Our results suggest that the combination of BET and MEK inhibitors is an effective therapeutic approach by modulating the epigenetic landscape for the treatment of ATC.

## RESULTS

### Combined treatment of JQ1 and trametinib totally inhibits cell proliferation in human ATC cells

**Figure 1:**
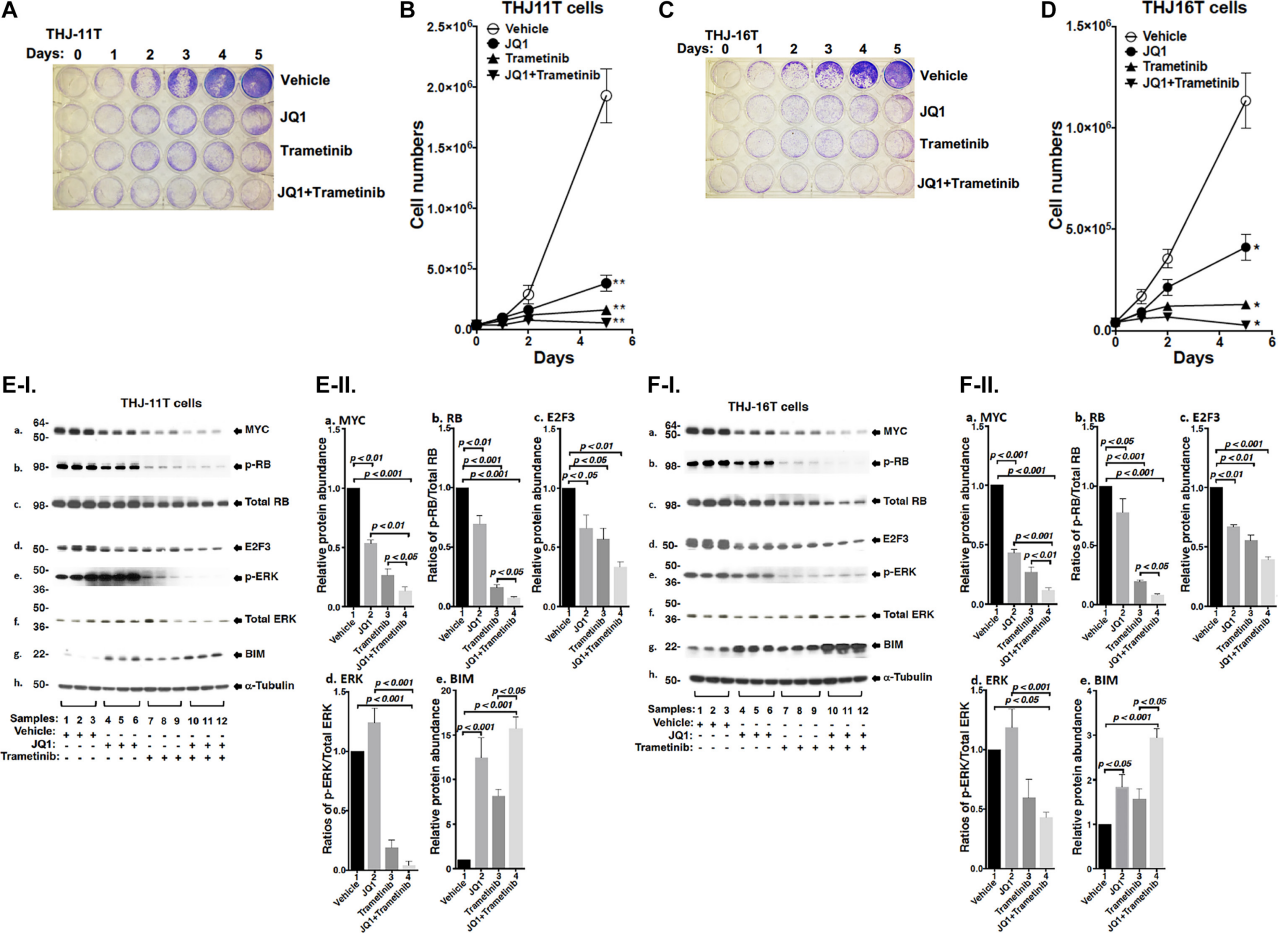
The combined treatment of JQ1 and trametinib completely blocked cell proliferation in the ATC cell lines (**A** and **C**) Cell proliferation visualized by crystal violet in the THJ-11T cells and THJ-16T cells, respectively. (**B** and **D**) Cell proliferation curves for the THJ-11T cells and THJ-16T cells, respectively. ^*^p < 0.05; ^**^p < 0.01. (**E** and **F**) Key cell regulators are affected by JQ1, trametinib, and the combined treatment. (**E-I**) and (**F-I**) are images of western blot analyses for MYC (a), p-RB (b), total RB (c), E2F3 (d), p-ERK (e), total ERK (f), BIM (g), and α-tubulin (h) as loading controls. Figure (**E-II**) and (**F-II**) are the quantification of the band intensities for the comparison among vehicle treatment, JQ1 treatment, trametinib treatment, and combined treatment with JQ1and trametinib in both cell lines (triplicates for each treatment with three independent experiments). The *p* values are indicated (*n* = 3).

We evaluated the effect of JQ, trametinib, and the combined treatment of the two on the proliferation of two human ATC cell lines: THJ-11T, THJ-16T. The authenticity of the THJ-11T and THJ-16T cell lines have previously been validated by DNA short tandem repeat analysis [20]. Thyroid specific markers in two cell lines were well characterized and matched to the originating tumors. They have been using for the screening of the drugs for the treatment of ATC. These two cell lines were derived from ATC patients that have different genetic lesions [20]. THJ-11T cells express a KRASG12V mutation, while THJ-16T cells express PI3KE454K, TP53, and Rb mutations. We found that either JQ1 (500 nM) or trametinib (500 nM) inhibited cell proliferation in both THJ-11T or THJ-16T cells (Figure 1A and 1C). The cell numbers in the THJ-11T cells were decreased by 78.3% and 90.6% after 5 days of treatment with JQ1 or trametinib, respectively (Figure 1B). The cell numbers in the THJ-16T cells were decreased by 62.4% and 89.5% after 5 days of treatment with JQ1 or trametinib, respectively (Figure 1D). However, combined treatment of JQ1 and trametinib completely inhibited cell proliferation in both THJ-11T or THJ-16T cells (Figure 1B and 1D, respectively). The findings indicated that combined treatment was most effective in the inhibition of tumor cell proliferation.

**Figure 2:**
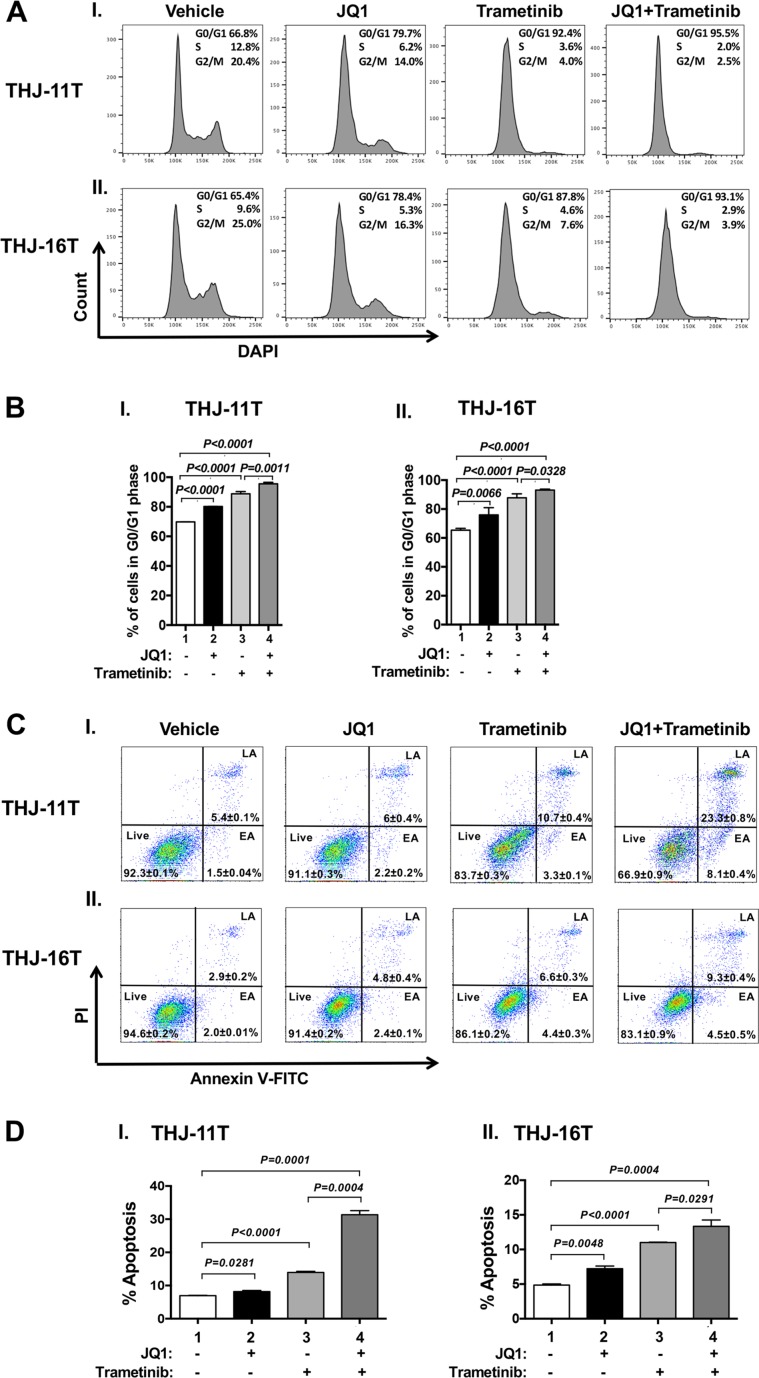
Effect of JQ1, trametinib, and the combined treatment on cell cycle progression (**A** and **B**) or apoptosis (**C** and **D**) of ATC cells by cytometric analysis. (**A**) Cell cycle profiles of THJ-11T (I) and THJ-16T cells (II). The average percentages of cell populations of different cell cycle phases were shown in the upper-right corner. (**B**) The graphs showed percent of G0/G1 phase for vehicle, JQ1, trametinib and combined treatment JQ1 with trametinib in THJ cell lines. Cell cycle phase was quantified by DAPI staining, followed by FACS analysis in THJ cell lines. Values are shown as means ± SD. (*n* = 3). (**C**). THJ-11T (I) and -16T cells (II) were treated with vehicle, JQ, trametinib, and both JQ1 and trametinib for 48 hours. After treatment, cells were collected and subjected to Annexin V and propidium iodide (PI) staining followed by flow cytometric analysis for apoptotic cells. (**D**) The populations of late and early apoptotic cells were quantified and graphed as shown in (I) and (II) for THJ-11T and -16T cells, respectively. The experiment was done in triplicates. Values are shown as means ± SD. (*n* = 3).

We next examined what cell regulators were affected by single or combined treatment, leading to the inhibition of tumor cell proliferation. Since JQ1 is known to inhibit the *MYC* transcription [9], we first examined the level of MYC protein and its downstream effectors. As shown in Figure 1E-I, panel a, we found that MYC protein levels in THJ-11T cells were lowered by JQ1 (lanes 4–6), trametinib (lanes 7–9), and the combined treatment (lanes 10–12). It is clear from the quantitation that the decreases were 46.5%, 73.5%, and 86.4%, respectively, by JQ1, trametinib, and the combined treatment (Figure 1E-II-a). The effect of the changes in MYC in cell proliferation was evident in that p-RB was reduced by JQ1 (panel b, lanes 4–6), trametinib (panel b, lanes 7–9), and the combined treatment (panel b, lanes 10–12, Figure 1E-I). Quantitation of ratios of p-RB versus total RB indicate that the combined treatment was the most effective (Figure 1E-II-b; 30%, 85%, and 93% for JQ1, trametinib, and the combined treatment, respectively). The protein levels of E2F3, the downstream effector of RB, were correspondingly lowered by the three treatments (Figure 1E-I-d and 1E-II-c). The decreased E2F3 protein levels would suggest that the cell cycle progression of ATC cells would be delayed. We therefore carried cell cycle analysis by flow cytomety. Figure 2A-I and -II show the profiles of G0/G1, S and G2/M cell cycles for THJ-11T and THJ-16T, respectively. The quantitative data indicated that the entry of ATC cells from the G0/G1 phase and S phase was significantly delayed (Figure 2B-I and -II, for THJ-11T and THJ-16T cells, respectively). Taken together, these data indicate that JQ1, trametinib, and the combined treatment decreased the proliferation of ATC cells by inhibiting the cell cycle progression and that the combined treatment was the most effective.

We next compared the effects of JQ1 and trametinib on the ERK signaling in THJ-11T cells. JQ1 did not affect p-ERK (Figure 1E-I-e and Figure 1E-II-d). However, as expected, p-ERK protein levels were markedly lowered by trametinib (Figure 1E-I-e, lanes 7–9), and further decreased by the combined treatment (lanes 10–12). The ratios of p-ERK versus total ERK were decreased 80% and 96% by trametinib and the combined treatment, respectively (Figure 1E-II-d). Bcl-2-like protein 11, commonly called BIM and a member of the BCL-2 protein family acting to promote apoptosis [21, 22], was reported to be a downstream effector of ERK signaling [23]. Accordingly, we assessed BIM protein levels in THJ-11T cells treated with JQ1, trametinib, and the combined treatment. Figure 1E-I-g shows that JQ1, trametinib, and the combined treatment elevated the BIM protein levels (Figure 1E-I-g) by 12.4-fold, 8.1-fold, and 15.7-fold, respectively (Figure 1E-II-e). These data suggested that the blocking of THJ-11T cell proliferation shown in Figure 1A by the combined treatment was mediated not only by inhibition of cell cycle progression, but also by increased apoptosis.

To validate the efficacy of combined treatment of JQ1 and trametinib on ATC cells, we also evaluated the effects of the two inhibitors on another ATC cell line, THJ-16T cells. As shown in Figure 1F-I, western blotting revealed that these inhibitors suppressed cell cycle progression by reducing MYC (panel a), decreased p-RB (panel b) and E2F3 (panel d). These inhibitors also attenuated the ERK signaling (panel e) to up-regulate BIM to promote apoptosis of THJ-16T cells (panel g). The quantitative changes in the cell regulators affected by JQ1, trametinib, and the combined treatment shown in Figure 1F-II indicate that the combined treatment had the highest efficacy in the inhibition of proliferation of THJ-16T cells. It is notable that the patterns of changes affected by JQ1, trametinib, and the combined treatment in THJ-11T (Figure 1E-I and 1E-II) and THJ-16T (Figure 1F-I and 1F-II) were similar, albeit with some minor quantitative differences.

That the pro-apoptotic regulator, BIM, was elevated in both the THJ-11T and THJ-16T cells (see Figure 1E-II-e and 1F-II-e) prompted us to functionally validate that, indeed, the double treatment was most effective in promoting tumor cell apoptosis to decrease cell growth. Flow cytometric analysis by cells stained with Annexin V and propidium iodide showed that JQ1, trametinib, and the combined treatment increased tumor cell apoptosis in both THJ-11T (Figure 2C-I) and THJ-16T (Figure 2C-II). Quantitative analysis showed that compared with vehicle-treated cells (bar 1, Figure 2D-I), 1.2-, 2.0-, and 4.5-fold increases of apoptotic cells in early apoptotosis (EA) and late apoptosis (LA) were detected in THJ-11T treated with JQ1, trametinib, and the combined treatment, respectively (Figure 2D-I, bars 2, 3, and 4). In THJ-16T cells, compared with vehicle-treated cells (bar 1, Figure 2D-II), 1.5-, 2.2-, and 2.7-fold increases of apoptotic cells (both EA and LA) were detected in cells treated with JQ1, tametinib, and the combined treatment, respectively (Figure 2D-II, bars 2, 3 and 4). These data further support that the combined treatment acted synergistically to promote tumor cell apoptosis.

### Combined treatment of JQ1 and trametinib inhibits growth of anaplastic thyroid tumors *in vivo* in xenograft mouse models

**Figure 3:**
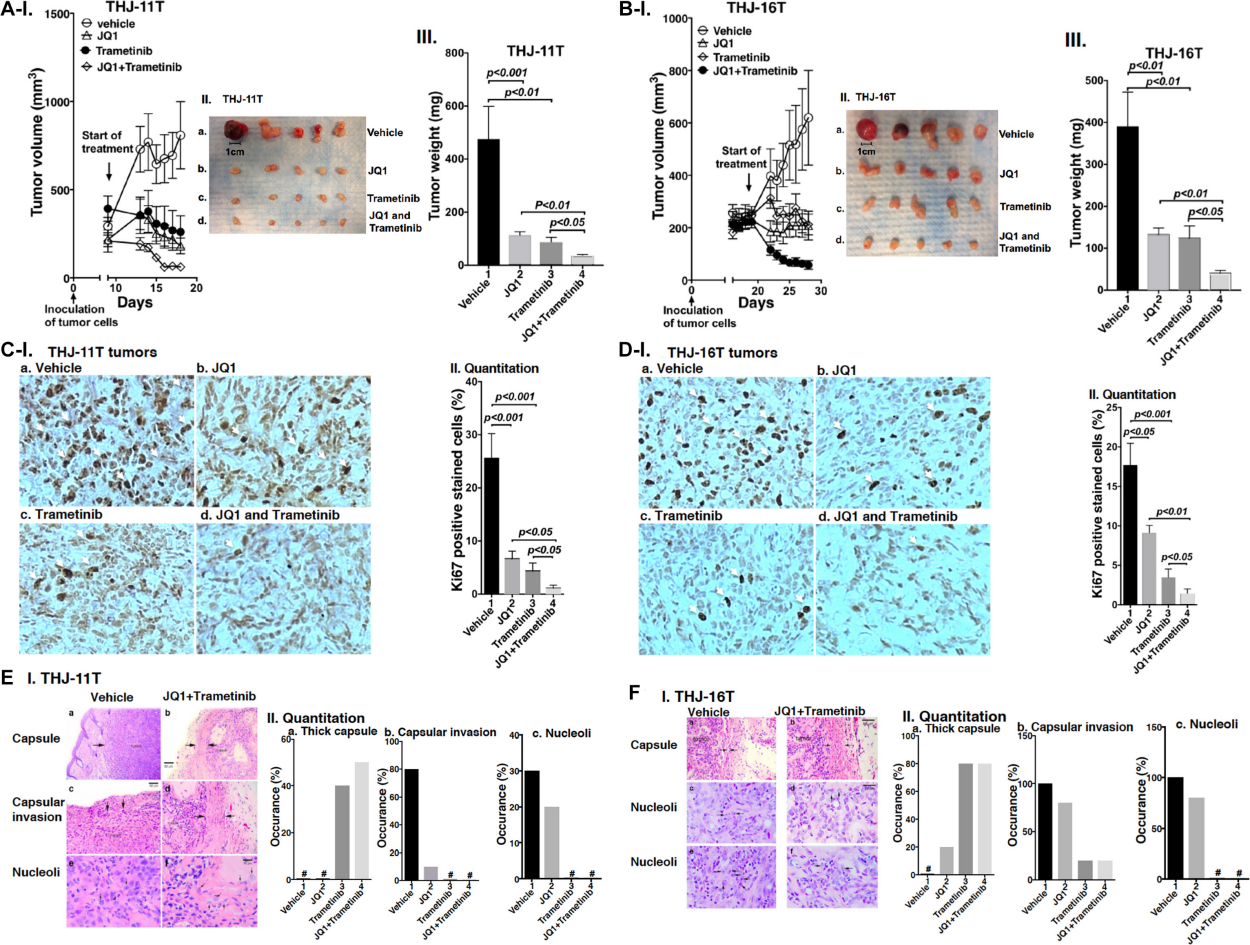
(**A–B**) The combined treatment of JQ1 and trametinib promoted tumor regression in anaplastic thyroid tumors in athymic mice. Equal numbers of THJ-11T cells (5 × 10^6^) or THJ-16T cells (5 × 10^6^) were injected into the flanks of mice before treatment. When tumors reached the size of 200 mm^3^, vehicle, JQ1, or trametinib was administered for 10 days. Tumor growth curves (I), tumor size (II) and tumor weight (III) for THJ-11T (**A**) and THJ-16T (**B**) are shown (*n* = 5 for each treatment). Ki67 staining of sections of tumor derived fromTHJ-11T cells (**C-I**) or from THJ-16T cells (**D-I**) as described in Methods. The treatment for each panel is marked. The Ki67 positively-labeled cells were counted, and % of Ki-67-positive cells is graphed in (**C-II** and **D-II**). The *p* values are indicated. (**E-I** and **F-I**). The H & E slides were prepared according to Methods. Representative pathohistological characteristics of xenograft tumors of ATC cells treated with vehicle (a, c, e) or with combined treatment (b, d, f) are marked. (**E-II** and **F-II**). % occurrence of capsular thickness (panel a), capsular invasion (panel b), and large nucleoli (panel c) were quantitified from H & E stained sections derived from THJ-11T cells (**E-II**, *n* = 5) and THJ-16T cells (**F-II**, *n* = 5). ^#^ denotes not detectable.

That combined treatment of JQ1 and trametinib inhibited ATC cell growth prompted us to test the efficacy of the inhibitors *in vivo* in mouse xenograft models. After THJ-11T cells were inoculated subcutaneously, tumor growth was visible after 2 days. When the tumor growth reached 200 mm^3^ (8 days after inoculation), we started the treatment (Figure 3A). Single treatment of JQ1 or trametinib blocked the tumor growth after treatment of 10 days (Figure 3A-I; also see Figure 3A-II and III). However, importantly, combined treatment further synergistically suppressed the tumor growth (Figure 3A-II and III). The extent of inhibition of the tumor growth induced by THJ-11T cells by the three treatments is shown in Figure 3A-III. The quantitative analysis indicated that 82%, 87%, and 92% of tumor growth, was suppressed by JQ1, trametinib, and the combined treatment, respectively.

The effectiveness of JQ1, trametinib, and the combined treatment was further evaluated in another ATC cell (THJ-16T). The induction of tumor growth by THJ-16T cells needed a longer time in that tumor growth reached 200 mm^3^ after 18 days as compared with 8 days for tumors induced by THJ-11T cells (Figure 3B-I). While the profile of the efficacy of JQ1, trametinib, and the combined treatment on the suppression of tumor growth induced by THJ-16T cells was similar to that of THJ-11T cells (compare Figure 3A-I and 3B-I), there were some minor quantitative differences in the extent of the inhibition of tumor growth. The extent of inhibition by JQ1, trametinib, and the combined treatment was 66%, 68%, and 88%, respectively (Figure 3B-III, bar 2, 3 and 4). The minor differences in the sensitivities might reflect that these two cell lines were derived from patients who had different genetic lesions [20]. Despite minor quantitative sensitivities to the inhibitors in both tumors, combined treatment resulted in synergistic suppression of tumor growth.

The effectiveness of the treatment by JQ1, trametinib, and the combined treatment was further analyzed by immnunohistochemical staining of Ki-67. In tumor cells from vehicle treatment mice, strong Ki67-postive cells were detected (Figure 3C-I, panel a). Treatment with JQ1 (panel b), trametinib (panel c), and the JQ1 and trametinib (panel d) markedly reduced the Ki67-positively stained cells. Quantitative analysis showed a 73%, 81%, and 95% reduction of Ki67-positively stained cells by JQ1, trametinib, and combined treatment (Figure 3C-II). Similar reductions in the positively Ki67 stained cells were also found in the tumors induced by THJ-16 cells (Figure 3D-I, compare panels b, c, d with a). Quantitative analysis indicated that compared with the vehicle-treated mice, the degree of reduction of Ki67 positively stained cells was 49%, 80%, and 92% for JQ1, trametinib, and combined treatment, respectively (Figure 3D-II). Taken together, these data indicated that in both tumors induced by either THJ-11T or THJ-16T cells, combined treatment was the most effective to inhibit tumor proliferation to suppress tumor growth.

We further analyzed the histopathological characteristics of the tumors induced by THJ-11T or THJ-16T cells. These tumors were examined for histopathology, and images of these changes with and without combined treatment are shown in Figure 3E-I for THJ-11T tumors, and in Figure 3F-I for THJ-16T tumors. The xenograft tumors induced by THJ-11T and THJ-16T showed dramatically decreased growth with treatment. In these figures, untreated tumors are shown in the left column, and tumors with combined treatment are shown in the right column. Vehicle-treated tumors had characteristic malignant morphology, including diminished capsular thickness (3E-I-a, 3F-I-a), capsular invasion by tumor cells (3E-I-c), and large eosinophilic nucleoli (3E-I-e, 3F-I-c and -e). Tumors with combined treatment showed thick capsules (between arrows) (3E-I-b, 3F-I-b). Minimal capsular invasion was seen in tumors with combined treatment (3E-I-d). Nucleoli in tumor cells with combined treatment were small and inconspicuous (see arrows in 3E-I-f, 3F-I-d and -f). These changes are quantitatively summarized in Figure 3E-II and 3F-II to indicate that compared with single treatment, combined treatment was the most effective to increase thick capsule (panels a), decrease capsular invasion (panels b), and decrease occurrence of large nucleoli (panels c).

**Figure 4:**
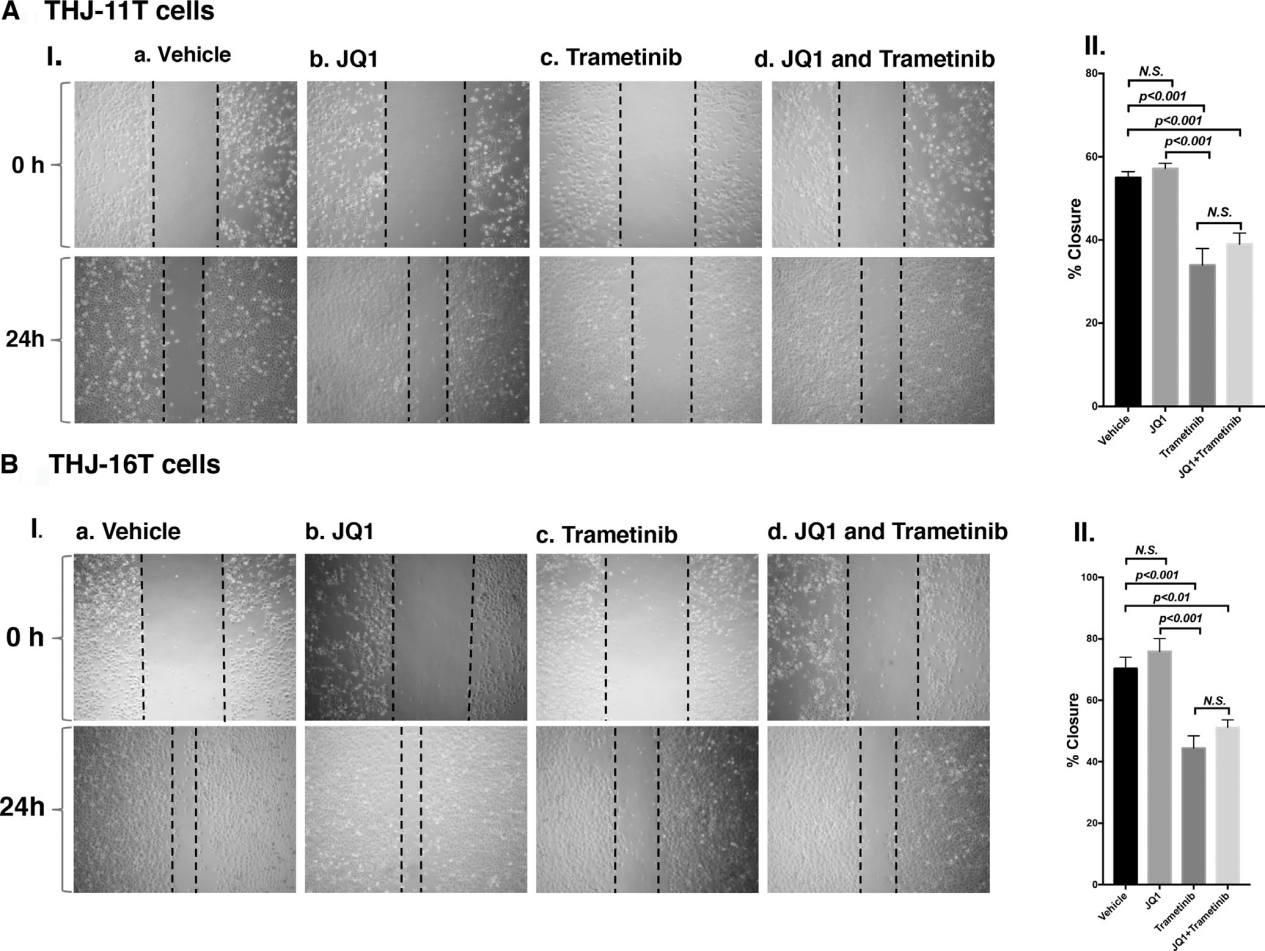
Trametinib and the combined treatment of JQ1 and trametinib inhibit ATC cell migration Monolayer scratch-induced migration assay. (**A-I**) THJ-11T cells or (**B-I**) THJ-16T cells treated with vehicle, JQ1, trametinib or combined JQ1 and trametinib were scratched with a 20-µl plastic pipette tip at 0 h and 24 h. (**A-II**) Quantitative analysis of wound-induced migration assay from (**A-I**). (**B-II**) Quantitative analysis of wound-induced migration assay from (**B-I**). The results are presented as mean ± SD of measurements of areas from six wells for each group.

To further support that the combined treatment suppressed tumor cell invasion/migration as shown in panel b (Figure 3E-II and 3F-II), we carried out cell migration assays. While JQ1 alone had little effect (Figure 4A-I-b, Figure 4B-I-b), trametinib and the combined treatment were effective in suppressing tumor cell migration (Figure 4A-II and 4B-II).

### Collaborative suppression of MYC and ERK signaling mediates growth inhibiton by the combined treatment in xenograft mouse models

**Figure 5:**
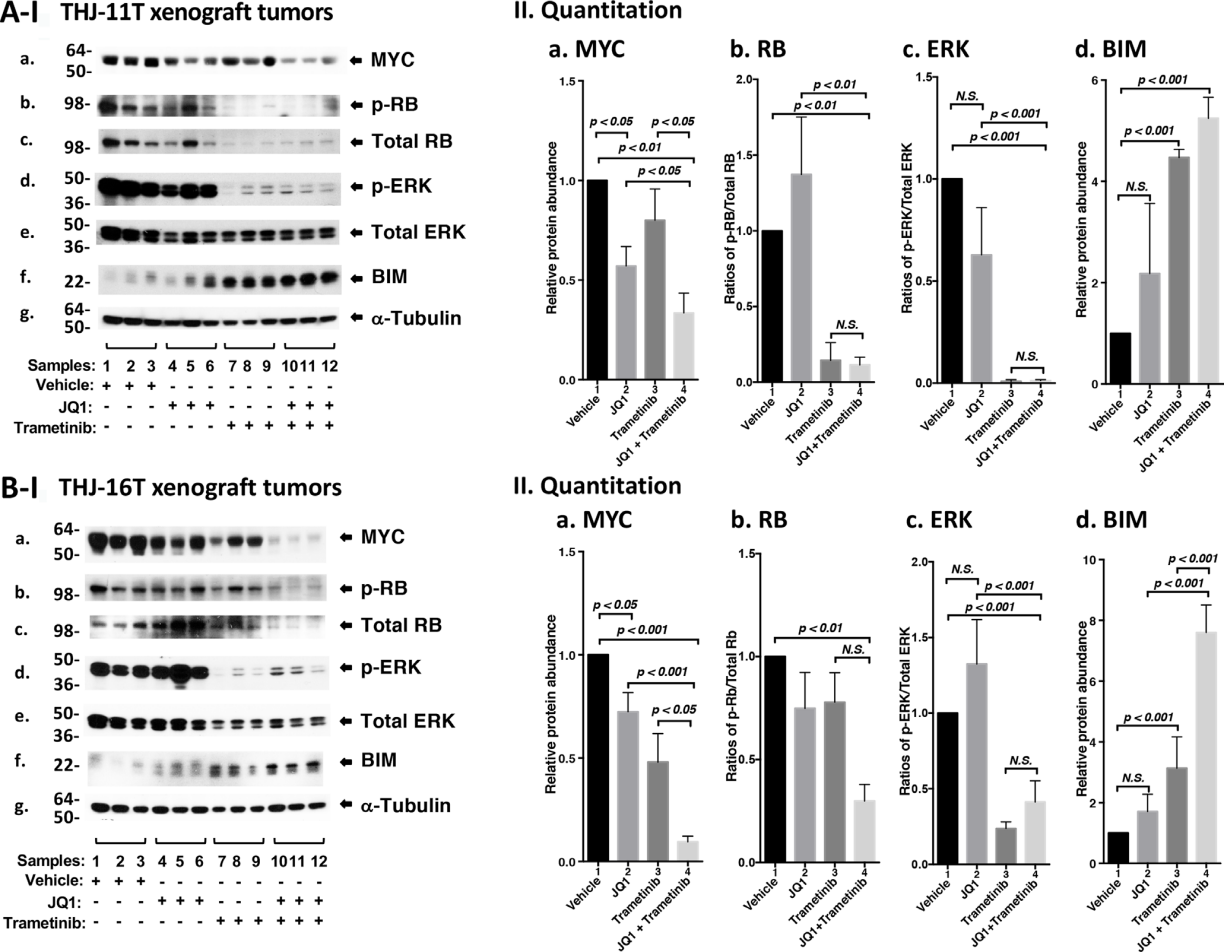
The combined treatment of JQ1 and trametinib synergistically decreased the abundance of *MYC* expression and altered downstream effectors in xenograft tumors (**A** and **B**) Total protein extracts were prepared from xenograft tumors induced by THJ-11T cells (**A**) and THJ-16T cells (**B**). (I) Western blot analysis was carried out for MYC (a), p-RB (b), total RB (c), p-ERK (d), total ERK (e), BIM (f), and α-tubulin (g) as loading control. (II) Band intensities in (I) were quantified for comparison among vehicle-, JQ1-, trametinib-, JQ1and trametinib-treated xenograft tumors. The experiments in each treatment were analyzed in triplicates with three independent experiments. The *p* values are shown (*n* = 3).

We next sought to identify the cell regulators affected by JQ1, trametinib, and the combined treatment, leading to the inhibition of xenograft tumor growth. Western blotting shows that JQ1 treatment decreased the MYC protein levels (Figure 5A-I, panel a, lanes 4–6) as compared with vehicle controls (lanes 1–3). Trametinib had no significant effect on the MYC protein level (lanes 7–9). However, the combined treatment synergistically lowered MYC by 70% (lanes 10–12, see also Figure 5A-II-a, bar 4). Trametinib as a single agent markedly decreased p-RB (panel b) and total RB with reduction in the ratios of p-RB versus total RB by 90% (Figure 5A-II-panel b, bar 3). With the strong effect on RB protein levels by trametinib, we could not detect further effect on RB protein level by the combined treatment (Figure 5A-II-b, bar 4). Trametinib, but not JQ1, lowered the p-ERK protein levels without changing the total ERK protein levels (Figure 5A-I-panels d and e). The ratios of p-ERK versus total ERK was reduced by single treatment as well as combined treatment by >95% (Figure 5A-II-panel c, bars 3 and 4), suggesting the critical contribution of MEK–ERK signaling cascade to the growth of xenograft tumors induced by THJ-11T cells. Trametinib increased the protein levels of BIM, a pro-apoptotic regulator (Figure 5A-I, panel f, and also 5A-II-d, bar 3). The combined treatment further elevated BIM (Figure 5A-I, panel f, lanes 10–12; and also 5A-II-d, bar 4). Take together, these data suggested induction of apoptosis by combined treatment also contributed to the inhibition xenograft tumor growth.

We also evaluated how the growth of xenograft tumors induced by THJ-16T cells was inhibited by different inhibitor treatment (Figure 5B). We found similar patterns of changes in MYC (Figure 5B-I-panel a), p-RB (panel b), p-ERK (panel d) and BIM (panel f) in the tumors induced by THJ-16T cells as tumors induced byTHJ-11T (Figure 5A-I). There were only some minor quantitative difference in the degree of changes such as in MYC, the synergistic effect of the combined treatment was more pronounced in the tumors induced by THJ-16T (Figure 5B-II-a, bar 4) than in tumors induced by THJ-11T (Figure 5A-II-a, bar 4). For BIM, the synergistic effect of double treatment was greater in the tumors induced by THJ-16T (Figure 5B-II-d, bar 4) than in tumors induced by THJ-11T (Figure 5A-II-d, bar 4).

### Convergent effects of JQ1 and trametinib suppress binding of BDR4 to the promoter of the *MYC* gene

**Figure 6:**
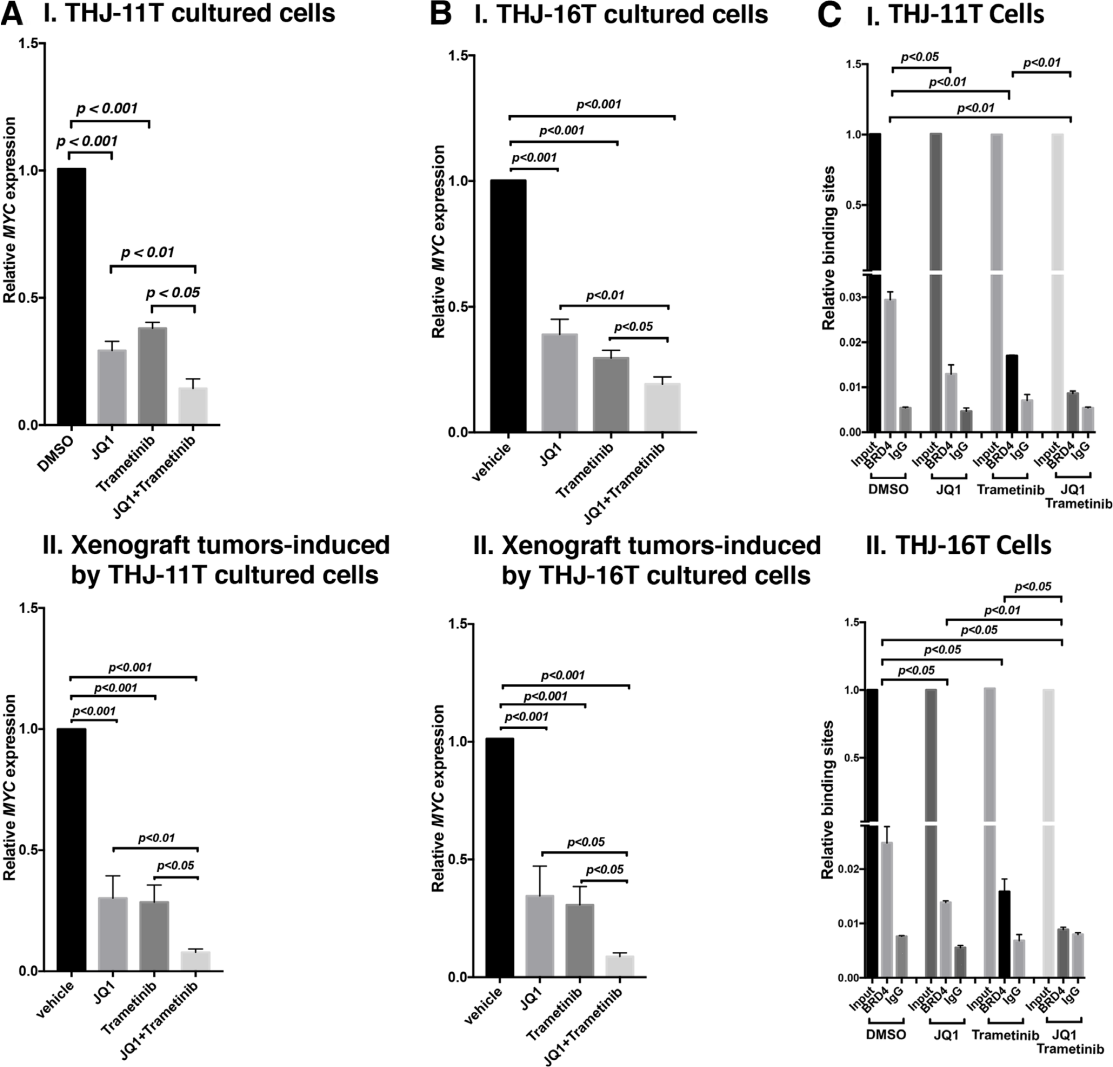
The combined treatment of JQ1 and trametinib synergistically decreased the *MYC* mRNA expression in ATC cell lines and tumors The mRNA level of the *MYC* gene was measured by real-time RT–PCR in THJ-11T cells (**A-I**) and in THJ-16T cells (**B-I**). The mRNA level of the *MYC* gene was measured by real-time RT–PCR in the THJ-11T xenograft tumors (**A-II**) and in the THJ-16T tumors (**B-II**). PCRs were performed using 200 ng of total RNA. *GAPDH* was used as a reference. The combined treatment of JQ1 and trametinib synergistically reduced the recruitment of BRD4 on the promoter of the *MYC* gene in THJ-11T cells (**C-I**) and in THJ-16T cells (**C-II**). ChIP assay was carried out using noremal rabbit IgG or BRD4 antibody. The experiments were carried out in triplicates. The *p* values are shown (*n* = 3).

That MYC protein levels were decreased by JQ1, trametinib, and combined treatment prompted us to examine the effect of inhibitors on the expression of *MYC* at the mRNA levels. Through inhibition of the binding of BRD4 to acetyl-lysines on chromatins, JQ1 is known to selectively inhibit transcription of the *MYC* gene in many tumors [9, 12, 24]. We found that the expression of *MYC* mRNA was inhibited by JQ1, trametinib, and the combined treatment by 70.8%, 62%, and 85.6%, respectively, in THJ-11T cells (Figure 6A-I). Consistently, in xenograft tumors induced by THJ-11T cells, the *MYC* mRNA expression was also similarly inhibited by JQ1, trametinib, and the combined treatment (69.8%, 71.4%, and 92.2%, respectively; Figure 6A-II). The extent of the inhibition of *MYC* mRNA expression was positively correlated with the MYC protein levels in that the combined treatment synergistically suppressed the expression of *MYC* at the mRNA and protein levels (see panel a of Figure 5A-I and 5B-I).

We also determined the MYC mRNA levels in THJ-16T cells. As found in THJ-11T cells, the expression of MYC mRNA was suppressed similarly in both the cultured THJ-16T cells *in vitro* by JQ1, trametinib, and the combined treatment (61%, 70.5%, and 80.9%, respectively, Figure 6B-I), and the xenograft tumors induced by THJ-16T cells (65.5%, 69.2%, and 91.2%, Figure 6B-II). These results indicate that, indeed, the JQ 1 and trametinib acted collaboratively to suppress MYC expression at the transcription level in both cultured cells and the xenograft tumors.

We next investigated the underlying mechanism by which JQ1 and trametinib acted collaboratively to suppress MYC expression. JQ1 functions as a competitive inhibitor with BRD4 for binding to the acetyl-lysines on the chromatin [12, 25]. We therefore carried out chromatin immunoprecipitation assay (ChIP) to evaluate the effects on the binding of BRD4 to the *MYC* promoter by the inhibitors in THJ-11T and THJ-16T cells. Compared with the control (vehicle-treated cells), 55%, 42%, and 69% inhibitions of the binding to BRD4 to the MYC promoter were detected in THJ-11T cells treated with JQ1, trametinib, and the combined treatment, respectively (Figure 6C-I). Similar profiles in the inhibition of binding to BRD4 by the three treatments in THJ-16T cells were detected (46%, 36%, and 64%, respectively, by JQ1, trametinib, and the combined treatment, Figure 6C-II). These results suggested that JQ1 and trametinib acted, at least in part, to suppress BRD4 binding to acetyl-lysines on the chromatin. Further, these results suggested that the inhibitory actions of JQ1 and trametinib could converge to collaboratively modify chromatin to suppress MYC transcription.

## DISCUSSION

Recent studies have shown that dysregulated epigenetic modifications, especially in early neoplastic development, may be just as critical as genetic mutations in driving cancer development and growth. The reversal of aberrant epigenetic alterations has therefore emerged as a potential strategy for the treatment of cancer. Dysregulated activation of *MYC* transcription by superenhancers on the chromatin has been shown to be a driver in the development of many cancers [26]. The discovery of specific inhibitors, such as JQ1, targeting the aberrant interaction of superenhancers with chromatin has proved effective in the treatment of hematological cancer and some solid tumors [10–13]. In line with these findings, we recently reported that in a mouse model of anaplastic thyroid cancer (*Thrb*^*PV/PV*^*Kras*^*G12D*^ mice), treatment of mutant mice with JQ1 inhibited thyroid tumor growth. However, JQ1 had no effect on the tumor invasion and metastasis in *Thrb*^*PV/PV*^*Kras*^*G12D*^ mice [16]. These findings suggested that thyroid tumor growth was MYC-dependent, whereas metastasis progression was driven by pathways other than MYC signaling in the ATC model of *Thrb* ^*PV/PV*^*Kras*^*G12D*^ mice. This notion would suggest that a combined treatment with another therapeutic, targeting the metastatic process could lead to a complete response. We therefore first tested this hypothesis in cell-based studies followed by *in vivo* studies using mouse xenograft models. We chose trametinib to combine with JQ1 in the treatment. Trametinib is a potent inhibitor of MAPK-ERK signaling and is currently being tested in several clinical trials to treat metastatic cancers [27]. We found that, indeed, combined treatment not only further enhanced the inhibition of tumor cell proliferation by a single agent, but also more effectively suppressed tumor cell invasion (see Figure 3E and 3F). These findings indicated the combination treatment, via targeting different pathways, could lead to synergistic effects for more effective treatment. This finding could be used as a rational basis in mapping out treatment modalities by targeting both growth and metastatic pathways for effective treatment of ATC.

In the present studies, we showed that the combined treatment of JQ1 and trametinib acted synergistically to inhibit tumor cell proliferation of THJ-11T and THJ-16T cells *in vitro* and to inhibit growth in *in vivo* xenograft tumors. We showed that JQ1 decreased the binding of BRD4 to the *MYC* promoter to suppress *MYC* transcription, leading to decreased tumor cell proliferation. Trametinib, a selective adenosine triphosphate-noncompetitive inhibitor of activation and kinase activity of MEK1 and MEK2 [28], blocked ERK phosphorylation. Via ERK downstream effectors, trametinib decreases cell proliferation, causes G1 cell cycle arrest, and induces apoptosis [29]. It is of importance to point out that the combined treatment of JQ1 and trametinib was most effective to induce apoptosis (see Figure 2C and 2D) in both both THJ-11T and THJ-16T cells. The proapoptotic BIM was most prominently up-regulated in both ATC cells (panels g in Figure 1E and 1F). The present findings are consistent with those reported earlier. Jing et al., found that the combined treatment of JQ1 and trametinib induced apoptosis of ovarian cancer cells via up-regulation of BIM [30]. Moreover, Paoluzzi et al., also found that the combined treatment of JQ1 together with vemurafenib, a BRAF inhibitor, effectively induced apoptosis of BRAF-mutant melanoma cells via up-regulation of pro-apoptotic regulator [31]. Thus, the present studies have demonstrated the broad effectiveness of the combined treatment of BETi and MEKi in many cancer types.

It is notable that trametinib also suppressed *MYC* transcription (see Figure 6) to lower MYC protein levels (Figure 1E, 1F and Figure 5). Moreover, ChIP analysis demonstrated that trametinib also acted to inhibit the binding of BRD4 to the *MYC* promoter (Figure 6C), suggesting that actions of JQ1 and trametinib could converge on chromatin by epigenetic modifications to suppress *MYC* transcription. At present, it is not known how trametinib inhibited BRD4 binding to the MYC promoter. One possibility is that trametinib exhibited JQ1-like activity in that it could compete with BRD4 for binding to acetyl-lysine on chromatin. That some kinase inhibitors could possess JQ1-like activity is without precedents. It was reported that the commonly used PI3-kinase probe LY294002 is an inhibitor of BET bromodomains [32]. It competitively inhibits acetyl-lysine binding of the first bromodomain of BET proteins [32]. Alternatively, it is also known that phosphorylation affects the stability of BRD4 and its interaction with the transcription mediator complex [33]. It is thus conceivable that trametinib could act as a kinase inhibitor to block the phosphorylation of BRD4 to attenuate the transcription of *MYC* via weakening of the interaction with transcription mediators. At present it is not known which of these two possibilities could account for the inhibition of binding to BRD4 to MYC by trametinib. However, the present studies uncovered a novel epigenetic action of trametinib to suppress *MYC* transcription, leading to inhibition of thyroid tumor growth.

The effect of combined treatment was assessed in two cell lines derived from ATC patients with different genetic lesions [20]. THJ-11T cells express KRASG12V mutation, whereas THJ-16T cells express PI3KE454K, TP53, and RB mutations. Interestingly, the THJ-16T cells with mutations in PI3KE454K, TP53, and RB proliferated more slowly than the THJ-11T cells that had only KRASG12V mutation (see growth curves in Figure 1B and 1D). Consistently, the development of xenograft tumors was also slower to reach 200 mm^3^ for THJ-16T cells (18 days, see Figure 3B) than THJ-11T cells (8 days, see Figure 3A). However, in spite of these differences, combined treatment seemingly had similar inhibitory effects on tumor growth induced by THJ-11T and THJ-16T cells, though with very minor quantitative differences (see Figure 3). Moreover, the extent of the changes of key cellular regulators affected by combined treatment was similar (Figure 1E versus 1F; Figure 5A versus 5B). These findings suggested that although the genetic lesions were distinct (KRASG12V mutation in THJ-11T cells and PI3KE454K, TP53, and Rb mutations in THJ-16 cells), aberrant signaling initiated by different upstream driver mutations would converge in the transcription responses. Thus, the components involved in the transcription machinery such as chromatin structure, transcription mediator, and polymerase complexes would be critical in the final manifestation of oncogenic responses. In support of this notion, recent global genomic analyses have demonstrated that in ATC, in addition to mutations in *TERT* promoter, *TP53, PI3K/AKT/mTOR* pathway effectors, mutations in SWI/SNF subunits, histone methyltransferases, and EIF1AX (a component of the translational preinitiation complex) were also detected [34]. In the fatal forms of non-anaplastic thyroid cancer, besides mutations in *TERT* promoter, *TP53, POLE, PI3K/AKT/mTOR* pathway effectors, high frequency mutations of *SWI/SNF* subunits, histone methyltransferases, *MED12* and *RBM10* were identified [35]. That high frequency of mutations detected for the chromatin modifiers (e.g., *SWI/SNF* subunits and histone methyltransferases) and transcription-associated regulators (e.g., MED12, *RBM10*, and EIF1AX) in ATC and fatal forms of non-ATC suggested the critical roles of chromatin/transcription regulators in the development of high-mortality thyroid cancer. These observations suggest that targeting chromatin/transcription regulators would be therapeutically beneficial. In support of this possibility, the present studies demonstrated that using JQ1, a prototype of BET inhibitors, synergistically enhanced by a MEK-specific inhibitor, was most effective in blocking proliferation and invasion of ATC *in vitro* and *in vivo*. With the development of a second generation of BET inhibitors and improved MAPK-MEK inhibitors, better dual treatment modalities could be identified and tested for efficacy in treating ATC, for which the current treatment options are limited.

## MATERIALS AND METHODS

### Cell culture

Two human anaplastic thyroid cancer cell lines (THJ-11T and THJ-16T) were gifts from Dr. John A Copland III at the Mayo Foundation for Medical Education and Research [20]. The cells were cultured in RPMI-1640 media supplemented with 10% fetal bovine serum (FBS), 1% non-essential amino acid, 1% sodium pyruvate, and 1% antibiotic-antimycotic solution (Thermo Fisher Scientific, Waltham, MA, USA) in 5% CO2 at 37°C in a humidified incubator.

### Cell proliferation assays

JQ1 was gifts from Dr. Jun Qi at the Dana Farber Cancer Institute, Harvard Medical School, Boston, Massachusetts, USA. Trametnib was purchased from the selleckchem (Houston, TX, USA). To evaluate the effect of inhibitors on cell proliferation, cells were cultured in the medium with JQ1, trametinib, or both JQ1 and trametinib at a concentration of 500 nM or DMSO in 6-well plates in triplicates. Cells were stained with crystal violet or counted daily for 5 days using a cell counter (Countess II, Thermo Fisher Scientific, Waltham, MA, USA).

### Western blot analysis

Western blot analyses were carried out as described previously [5]. Tumor tissues or cultured cells were washed with phosphate-buffered saline and homogenized in a solution with 50 mM Tris buffer, 150 mM NaCl, 1 mm ethylenediaminetetraacetic acid, 1% NP40 and proteinase (Roche Diagnostics, GmbH, Mannheim, Germany)/phosphatase inhibitors (Halt Phosphatase inhibitor cocktail, Thermo Scientific). After centrifuge at 14000 rpm for 5 minutes, lysates were used for Western blot analysis. The antibodies against MYC were used (ab32072) from Abcam, p21 (sc-6246), E2F-3 (sc-878) from Santa Cruz Biotechnology, INC (Santa Cruz, CA, USA), phosphorylated RB (S780) (#9307), RB (#9309), Bim (#2933), p-Erk1/2 (#9101) and total-ERK (#9102) from Cell Signaling, alpha tubulin (T6199) from Sigma Aldrich. After being washed, the membranes were incubated with horseradish peroxidase-conjugated anti-mouse immunoglobulin G [21] (NA9310 GE Healthcare) or anti-rabbit IgG (NA9340 GE Healthcare) as the secondary antibody and subsequently detected by means of an ECL system (Western Lightning^®^ Plus-ECL, PerkinElmer, Waltham, MA, USA). Band intensities were quantified by the ImageJ software (ImageJ 1.48v; Wayne Rasband, NIH).

### Histological analysis

Immunohistochemistry was performed on formalin-fixed paraffin tumor sections as described previously [16]. Primary antibodies used were anti-Ki-67 antibody (Thermo Scientific, Fremont, CA, USA; #RB-9043-P0). Staining was developed with diaminobenzidine (DAB) using the DAB substrate kit for peroxidase (Vector Laboratories, Burlingame, CA, USA, SK-4100). IHC was carried out in triplicates. Five fields of cells positively stained with anti–Ki-67 antibody or without anti–Ki-67 antibody were randomly selected for counting from each slide. The percentage of the positively stained cells was calculated by a formula [(number of the positively stained cells/the number of total cells)x100%].

### RNA extraction and real time RT-PCR analysis

RNA extraction and real time RT-PCR analysis were carried out as previously described [16]. For the MYC gene: forward, 5′-CCTACCCTCTCAACGACAGC-3′; reverse, 5′-CTCTGACCTTTTGCCAGGAG-3′. For the glyceraldehyde-3-phosphate dehydrogenase (GAPDH) gene: forward, 5′-CGAGATCCCTCCAAAATCAA-3′; reverse, 5′-GGTGCTAAGCAGTTGGTGGT-3′.

### *In vivo* xenograft tumor assays

For xenograft studies, 6- to 8-week-old female athymic nude mice were used. All animal experiments were performed under protocols approved by the National Cancer Institute Animal Care and Use Committee. Five million of the THJ-11T or THJ-16 cells in 200 μl 50% Matrigel basement membrane matrix (BD Biosciences, Cat. 354234) were inoculated subcutaneously into the right flank of mice 2 to 3 weeks before treatment. The treatment with vehicle, JQ1, trametinib, or both JQ1 and trametinib started when the median tumor size reached approximately 200 mm^3^. JQ1 was administered via intraperitoneal injection at a dose of 50 mg/kg/mouse per day. Trametinib was administered via oral gavage at a dose of 1 mg/kg/mouse per day. The tumor size was measured with a caliper every 1–4 days. After about 10 days of treatment, all mice were euthanized and the tumors were dissected for further analysis.

### Flow cytometric analysis of apoptosis

THJ-11T and -16T cells were treated with vehicle, JQ (500 nM), trametinib (500 nM), and both JQ1 and trametinib for 48 hours. After treatment, cells were collected and subjected to Annexin V and propidium iodide (PI) staining using an FITC-Annexin V Apoptosis Detection Kit I (Cat#556547, BD Bioscience, San Jose, CA, USA), according to the manufacturer’s instructions. Apoptotic cells were then analyzed by flow cytometry (LSRfortessa II, BD Bioscience, San Jose, CA, USA) and analyzed with FlowJo (FlowJo LLC, Ashland, OR, USA).

### Flow cytometric analysis of the cell cycle

THJ-11T and -16T cells were treated with JQ1(500 nM) and/or trametinib (500 nM) for 48 h. Control cells were treated with DMSO. After treatment, cells were collected and fixed with cold 70% (vol/vol) ethanol and stored at –20°C at least 2 hours. After then, cells were washed in PBS buffer and then incubated with 1 µg/ml DAPI (4′, 6-diamidino-2-phenylindole) solution for 10 min before being analyzed. The cell cycle profiles were determined using flow cytometry (LSRfortessa II, BD bioscience, San Jose, CA, USA) and analyzed with FlowJo (FlowJo LLC, Ashland, OR, USA). All experiments were performed in triplicates.

### Migration assays

For monolayer scratch-induced migration assays, THJ-11T cells or THJ-16T cells were seeded in 6-well plates at a density of 3×10^5^ cells/well and grown until they reached a confluence of ∼70%. A scratch was made using a sterile 20-μl pipette tip for each well of the cells treated with vehicle, JQ1, trametinib or both of JQ1 and trametinib. Images of cells with a scratch were captured at the time of the scratch (0 h) and 24 h after scratch. The percentage of closure was calculated using a formula of [((pre-migratio area)-(migration area))/(pre-migration area)x100%]. The experiments were repeated three times and representative pictures from 6 wells are shown.

### Chromatin immunoprecipitation (ChIP) assay

ChIP assay was performed with slight modification as described previously [36]. Briefly, cultured cells treated with DMSO, JQ1, trametinib or combined JQ1 and trametinib were fixed in 1% of formaldehyde for 10 min and quenched by addition of glycine with a 0.125 M final concentration for 5 min. Immunoprecipitation was carried out using Anti-BRD4 (A301-985A50, Bethyl Laboratories Inc) or IgG as a negative control. The primers for the *MYC* gene were forward, 5′- GAGCAGCAGAGAAAGGGAGA-3′; and reverse, 5′- CAGCCGAGCACTCTAGCTCT-3′.

### Statistical analysis

All data are expressed as mean ± standard deviation. All tests were two-sided and *p* < 0.05 was considered significant. GraphPad Prism version 5.0 for Mac OS X was used to perform analyses of variances. All *in vitro* experiments were carried out in triplicates. The xenograft experiments with 5 biological samples from the THJ-11T-induced tumors were repeated three times. The xenograft experiments with 5 biological samples for the THJ-16T cells-induced tumors were repeated two times.
